# Laser-Induced Breakdown Spectroscopy for Rapid Discrimination of Heavy-Metal-Contaminated Seafood *Tegillarca granosa*

**DOI:** 10.3390/s17112655

**Published:** 2017-11-17

**Authors:** Guoli Ji, Pengchao Ye, Yijian Shi, Leiming Yuan, Xiaojing Chen, Mingshun Yuan, Dehua Zhu, Xi Chen, Xinyu Hu, Jing Jiang

**Affiliations:** 1 Department of Automation, Xiamen University, Xiamen 361005, China; glji@xmu.edu.cn (G.J.); 23220160154002@stu.xmu.edu.cn (P.Y.); msyuan@stu.xmu.edu.cn (M.Y.); 2 Innovation Center for Cell Signaling Network, Xiamen University, Xiamen 361102, China; 3 College of Physics and Electronic Engineering Information, Wenzhou University, Wenzhou 325035, China; yuan@wzu.edu.cn (L.Y.); 00101094@wzu.edu.cn (X.C.); 20150002@wzu.edu.cn (X.C.); 20170059@wzu.edu.cn (X.H.); 4 College of Mechanical and Electrical Engineering, Wenzhou University, Wenzhou 325035, China; zhudehua@wzu.edu.cn; 5 LifeFoundry, Inc., Champaign, IL 61820, USA; 6 Department of Electrical and Computer Engineering, University of Illinois at Urbana-Champaign, Urbana, IL 61801, USA

**Keywords:** toxic heavy metal, laser-induced breakdown spectroscopy (LIBS), *Tegillarca granosa*, discrimination analysis, wavelet transform algorithm (WTA)

## Abstract

*Tegillarca granosa* samples contaminated artificially by three kinds of toxic heavy metals including zinc (Zn), cadmium (Cd), and lead (Pb) were attempted to be distinguished using laser-induced breakdown spectroscopy (LIBS) technology and pattern recognition methods in this study. The measured spectra were firstly processed by a wavelet transform algorithm (WTA), then the generated characteristic information was subsequently expressed by an information gain algorithm (IGA). As a result, 30 variables obtained were used as input variables for three classifiers: partial least square discriminant analysis (PLS-DA), support vector machine (SVM), and random forest (RF), among which the RF model exhibited the best performance, with 93.3% discrimination accuracy among those classifiers. Besides, the extracted characteristic information was used to reconstruct the original spectra by inverse WTA, and the corresponding attribution of the reconstructed spectra was then discussed. This work indicates that the healthy shellfish samples of *Tegillarca granosa* could be distinguished from the toxic heavy-metal-contaminated ones by pattern recognition analysis combined with LIBS technology, which only requires minimal pretreatments.

## 1. Introduction

Due to the recent accelerated process of industrialization in developing countries, a large number of toxic heavy metals are discharged into rivers, lakes, and seas. The toxic heavy metal pollution of aquatic products has become an increasingly serious issue. Toxic heavy metals not only disrupt the living condition of aquatic animals in natural water resources but also intoxicate or kill aquaculture fish, which turns out to be a threat to the fish farming industry. Furthermore, heavy metal ions accumulate in the human body from the consumption of the polluted seafood [1,2]. Commonly, many enzymes in the human body will be deactivated by heavy metals, leading to the risk of chronic poisoning. Therefore, it is necessary to improve the detectability of heavy-metal-polluted aquatic products in order to ensure safety of consumers.

In order to study the pollution from heavy metal ions, we chose *Tegillarca granosa* as our subject of study. *Tegillarca granosa*, also well known as the blood clams of arcidae, is a kind of wide-temperature bivalve mollusk that perches on shallow and soft mudflats. It is one of the most important commercial seafood products in East Asia because it is delicious and nutritious [3,4]. Due to its low activity, filter-feeding habits and wide-distribution in coastal regions, which usually suffer from heavy pollution, *Tegillarca granosa* has a strong biological tendency to accumulate heavy metals. The heavy metals are directly absorbed from the surrounding water through the gills of *Tegillarca granosa*. Consequently, the adsorbed heavy metal ions are either transported to the whole body of *Tegillarca granosa* through the bloodstream or accumulated in the cell membrane [5]. Some heavy metal ions are easy for *Tegillarca granosa* to discharge, whereas others may stay and damage its tissue structure. Because of the environmental persistence of the chemicals, *Tegillarca granosa* tends to concentrate heavy metal ions from its food chains, which shows the potential of becoming the standard eco-toxicological subjects [6,7]. Therefore, the detection of heavy metal pollution using *Tegillarca granosa* sample is significant in eco-toxicological assessments.

Traditional heavy metal ion detection methods have their own limitations such that they cannot achieve cost-effectiveness, high sensitivity, easy-to-use, and destructive-free at once. For example, the chemical detection methods including flame atomic absorption spectroscopy [8], inductively coupled plasma-mass spectrometry [9], graphite furnace atomic absorption spectrometry [10], and the electrochemical method [11,12] are well-known for high detection sensitivity and superior accuracy. However, they are expensive, sample-destructive, and require experienced and skilled technicians to perform measurement. The biological detection methods using biosensors and enzyme-linked immunosorbent assays are portable, simple, and highly sensitive, but require a tedious sensor preparation process [13,14]. Alternatively, spectroscopic methods have also been applied to the detection of heavy-metal-contaminated aquatic products [15,16,17,18]. They are fast, accurate, and pollution-free, requiring minimal sample pretreatment. Thus, spectroscopic methods are better candidates for the rapid detection of heavy metal concentrations in *Tegillarca granosa*. Among different spectroscopic methods, laser-induced breakdown spectroscopy (LIBS) is a spectroscopic technique that can in principle detect any heavy metal elements regardless of their physical states [19]. This technique is mainly based on the one-to-one correspondence relationship between the specific elements and the characteristic wavelengths of atomic/ionic spectra. When the plasma induced by lasers on the sample surface cools down, spectra conveying the component and element information of the samples can be collected. Therefore, the laborious and time-consuming sample pretreatment required by other methods are not indispensable for the LIBS technique. As a fast and convenient technology for field detection, LIBS can be applied effectively to substitute other traditional methods for elemental detection and analysis. Moreover, the hand-held LIBS equipment is also commercially available. Thus, this technique is a suitable candidate for on-site testing, which can be applied in industrial analysis [20,21], environment monitoring [22,23,24], food safety [25,26], cultural heritage [27,28], biomedical analysis [29,30], and space exploration [31,32].

However, in real application, the LIBS spectra of real-life samples like *Tegillarca granosa* are too complicated to be analyzed by humans directly because the samples contain abundant elements and complex compositions. To make things worse, some characteristic spectral lines of different metals overlap due to mutual interferences of different chemicals. These have prevented the general public from applying advanced sensing tools to the benefit of their own lives for a long time. Fortunately, as pattern recognition algorithms develop, some modern computational tools can be adopted to analyze spectra data in sensing applications. Due to the limited information shown by the original LIBS spectra, efficient data mining methods are needed to extract characteristic spectral information as well as suppressing the interferences and noises [33]. Our work is a proof of concept study aiming at identifying a suitable machine learning algorithm to distinguish polluted *Tegillarca granosa* from safe ones.

In this study, several types of *Tegillarca granosa*, including uncontaminated control samples, samples contaminated by cadmium (Cd), zinc (Zn), lead (Pb), and those contaminated by a mixture of all three heavy metals, were evaluated with a combination of LIBS technology and discrimination analysis with pattern recognition. The specific objectives were as follows: (1) to decompose original LIBS spectra via the wavelet transform algorithm (WTA); (2) to extract the characteristic information from the high frequency coefficients of the wavelet transform domain using the information gain algorithm (IGA); (3) to identify *Tegillarca granosa* samples contaminated by a certain metal or multiple heavy metals using the extracted characteristic information as the input of discrimination models.

## 2. Materials and Methods

### 2.1. Sample Preparation

*Tegillarca granosa* samples, provided by Zhejiang Mariculture Research Institute (Wenzhou, China), were acclimatized to the laboratory conditions for approximately 10 days in plastic pools with cubic dimension of 60 cm × 40 cm × 30 cm. Analytical-grade PbCH_3_COO•3H_2_O, CdC1_2_, and ZnSO_4_•7H_2_O were purchased from the Chemical Reagent Co. Ltd., Shanghai, China.

After an over 24 h sedimentation process, the seawater was then filtered to remove sands for raising the *Tegillarca granosa* in tanks. The parameters of the seawater were a pH of 8.05 ± 0.10, a temperature of 22.4 ± 5.6 °C, a dissolved oxygen content of >6 mg/L, and a salinity level of 21%. Throughout the experiment, the seawater was exchanged every 24 h, after which the containers were refilled and dosed with the metal toxicant.

A total of 150 *Tegillarca granosa* samples were randomly divided into five equal size groups, i.e., 30 for each group. The *Tegillarca granosa* samples in Groups I, II, and III were exposed to water dissolved with highly concentrated PbCH_3_COO•3H_2_O (1.833 mg/L), CdC1_2_ (1.634 mg/L), and ZnSO_4_•7H_2_O (4.424 mg/L), respectively. Group IV was exposed to a mixture of equal amounts of the above three chemicals. Group V (control) was raised in the seawater without any spiked solution of heavy metal ions. After 10 days, which allowed the heavy metals to accumulate in the sample, they were sacrificed and kept in a refrigerator at −4 °C for 30 min. The samples were freeze-dried for subsequent spectral measurement.

### 2.2. Spectral Collection

**Figure 1:**
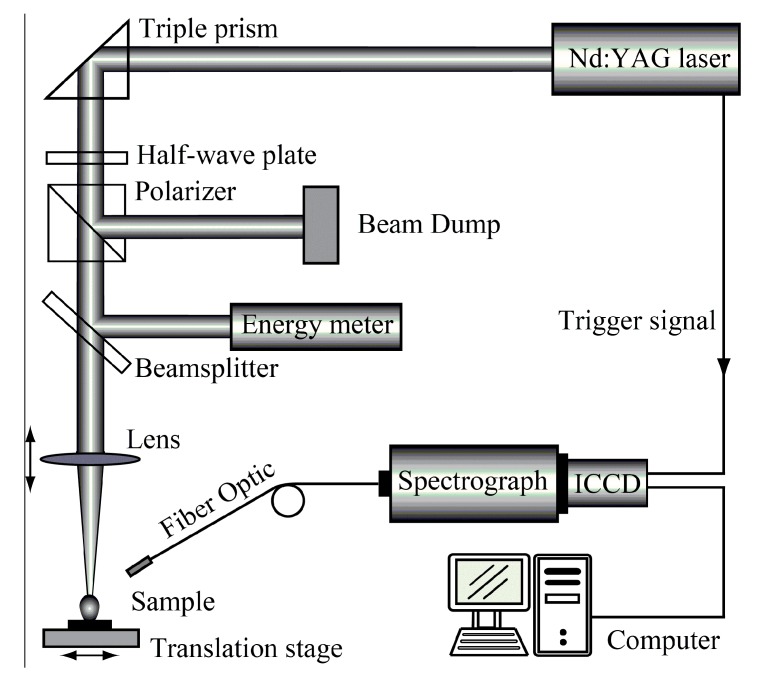
Schematic diagram of the laser-induced breakdown spectroscopy (LIBS) set-up.

The experimental set-up is shown in Figure 1. Each sample was measured five times at different spots on the translational stage. A pulsed Nd:YAG laser (Litron Nano SG 150-10, Litron Lasers, Warwickshire, England) with a wavelength of 1064 nm, a pulse duration of 6 ns, and an energy of 150 mJ was applied. A plano-convex lens (f = 100 mm) was used to focus the laser beam onto the sample surface at normal incidence. The spot diameter on the sample surface was set at 500 μm to improve the detection accuracy of contamination at certain local areas. A beam splitter was utilized to split a small fraction of energy (10%) for monitoring the pulse energy by an energy meter.

The plasma emission was collected using an optical fiber system at a 45° angle to the incident laser beam, which was then fed into a spectrometer (LTB Aryelle 150, Berlin, Germany) equipped with an optical chopper (with a time resolution of 0.1 µs). The spectral resolution was 6000 nm/nm. A charge coupled device (CCD) camera was used for the spectra acquisition, and the laser was synchronized with the spectrometer. The CCD gate width was 30 µs. The delay from the laser pulse generation to the start of spectral acquisition was set to 1 μs in all experiments.

### 2.3. Wavelet Transform Algorithm

The wavelet transform algorithm (WTA) is a time–frequency localization analysis method that exhibits excellent performance in the analysis and extraction of non-stationary signal characteristics [34]. In WTA, the scaling and translating operations on the signal gradually achieve multi-scale refinement, which eventually leads to appropriate time resolutions of high frequency signals and remarkable frequency resolutions of low frequency signals. WTA can automatically adapt to the requirements of the time–frequency signal analysis, so as to focus on any signal details of interest. This method provides an alternative to Fourier transform. WTA has been successfully applied in many fields, including medical science and image processing [35,36]. The Wavelet toolbox in Matlab 2012a is used in this work.

### 2.4. Information Gain Algorithm

In information theory, entropy denotes the average unpredictability of a random variable, which is considered to be equivalent to the content of the information. The information gain algorithm (IGA) can effectively select the important features based on entropy. The expected value of information gain (IG) is the mutual information of the target variables and the independent variables. The reduction in the entropy of one target variable is achieved by learning the state of the independent variable. IGA treats each feature in isolation and estimates how important it is for the prediction of the correct class label. The entropy-based attribute selection and ranking method aims to minimize the entropy value of an attribute, thereby maximizing its IG. The main advantage of this method is that it includes all attributes in the analysis [37,38].

### 2.5. Spectral Calibration and Analysis Methods

Samples were randomly divided into two subsets (i.e., calibration and prediction subset) with the ratio of 4:1. In this way, 120 samples in the calibration subset were used to train discrimination models, and the other 30 samples in the prediction subset were to test the accuracy of model. The target was encoded in binary vector format such as 1000, 0100, 0010, and 0001 to represent different contamination types of the samples for discrimination analysis. Here, three extensively used classifiers, Partial least squares discriminant analysis [39,40,41] (PLS-DA), Random Forest [42] (RF), and least squares support vector machine [43,44] (LS-SVM), were employed to calibrate a discrimination model.

## 3. Results and Discussion

### 3.1. Analysis of LIBS Spectra

**Figure 2:**
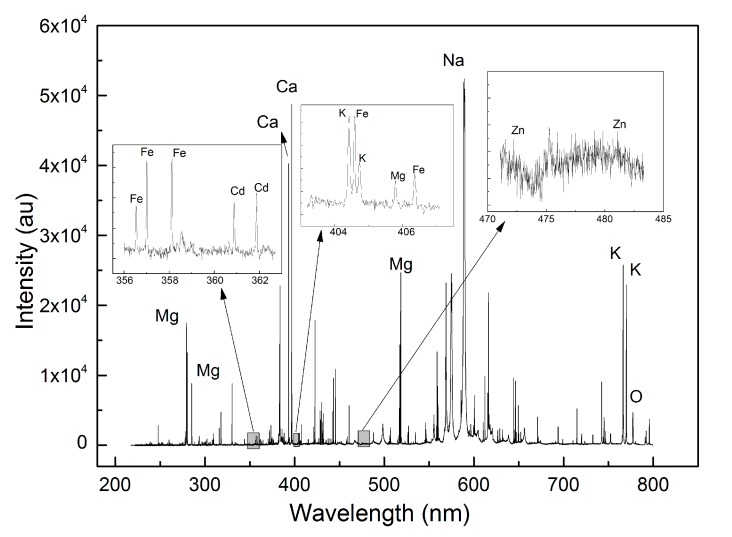
Typical LIBS spectra of Group IV *Tegillarca granosa* samples.

The representative LIBS spectra for Group IV samples in the wavelength region of 200–900 nm is shown in Figure 2. In general, the LIBS spectra for *Tegillarca granosa* samples were quite complex, containing multiple peaks contributed by different elements. The acquired LIBS spectra have distinct elemental features. These profiles in Figure 2 contain atomic and ionic lines of Al, C, Ca, Cd, Pb, Fe, K, Mg, Na, Si, Sr, and Zn, spreading over the wavelength range. The emission lines with high intensity were Ca I (422.7 nm), Ca II (393.3 nm, 396.8 nm), Na I (588.9 nm, 589.5 nm), Mg I (285.2 nm; 517.3 nm; 518.4 nm), Mg II (279.5 nm; 280.3 nm), and K I (766 nm; 770 nm). Less prominent emission lines, including C I (247.8 nm), Sr I (460.7 nm), Zn I (330.3 nm), Si I (288.2 nm), Al I (394.4 nm; 396.2 nm), and Fe I (438.4 nm; 440.5 nm), were also observed. Although some characteristic peaks in the LIBS spectra of *Tegillarca granosa* are remarkable, heavy metals like Zn and Pb are greatly interfered by the noises. As a result, samples in the control group cannot be directly distinguished from the ones from heavy-metal-contaminated groups. Therefore, machine learning algorithms were required to classify the corresponding group for each sample.

### 3.2. Analysis of Discrimination Results Using Full Spectra

PLS-DA algorithm was employed to extract the information of predictive variables hidden in complex spectral information for achieving the purpose of compression and information extraction. A discrimination model was built based on the full spectral information being regarded as the input variables for the PLS-DA model. Unfortunately, this method was ineffective, providing less than 30% accuracy for validation samples. A few factors may have resulted in the poor discrimination performance: (1) the full spectra contain high noise or irrelevant information, while the characteristic information is less evident between the different contaminated *Tegillarca granosa*; (2) serious mutual interference from laser pulses and heterogeneous matrix effect could have reduced the quality of the LIBS spectra; (3) PLS-DA, which is generally capable of processing strongly linear problems, is not the best candidate to predict the group of the samples with strong nonlinearity between LIBS spectra and predictive variables [19]. To solve the nonlinearity problems of the LIBS spectra, additional tools working for nonlinear models, including RF and SVM, were adopted to discriminate the different types of *Tegillarca granosa*. However, the accuracy was as low as 40%, possibly because the useful characteristic information may have been submerged in the noise. From this study, we concluded that further data mining methods were indispensable to extract the characteristic bands from the original full spectral information.

### 3.3. Analysis of Results Using Characteristic Spectra

**Figure 3:**
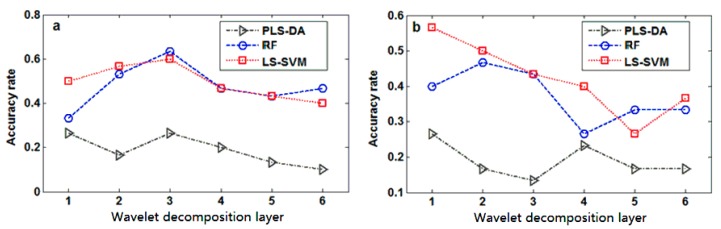
Recognition results at different decomposition layers: (**a**) high frequency components; (**b**) low frequency components.

**Figure 4:**
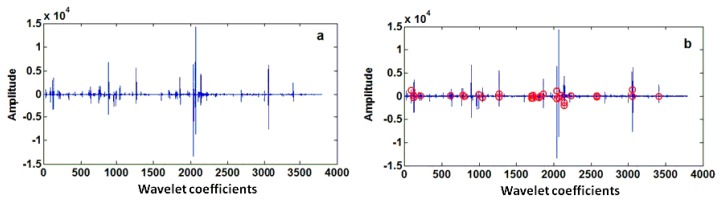
(**a**) Wavelet coefficients of the third decomposition level; (**b**) Characteristic variables in the wavelet coefficients of the third decomposition level (red circles represent the characteristic variables extracted by the information gain algorithm (IGA)).

The LIBS spectra showed that the characteristic peaks from most elements were narrow and very similar to the “high frequency” signals. Based on these characteristics, the discrete wavelet transform (DWT), an algorithm widely applied to time–frequency analysis of signals, was adopted for further spectra analysis. This algorithm can decompose the ‘‘high’’ and ‘‘low frequency’’ parts of the original signals, so that we can apply two different filters to access the different frequency components of the signals. The widely used Daubechies 4 (Db4) wavelet was adopted as the kernel function of DWT to transform the original LIBS spectra. The high frequency components from different decomposition layers were then extracted and taken as the input variables to develop PLS-DA, RF, and LS-SVM discrimination models. The classification results from the high frequency components of different decomposition layers are shown in Figure 3a. Because a few metal elements have multiple excitation states, the contamination category of *Tegillarca granosa* samples is nonlinear with the spectral lines. The PLS-DA model applied in the different decomposition layers showed an accuracy similar to that shown by the model applied in the full spectra. Compared with PLS-DA, RF and LS-SVM performed slightly better because they can process nonlinear models more effectively. The recognition results analyzed by RF and LS-SVM methods from the first six decomposition layers significantly varied, showing a serious fluctuation in the discrimination accuracy in the prediction dataset. This may be because more characteristic information (i.e., high frequency information) is extracted when the number of decomposition layers increases. The discrimination ratio reached its highest level at the decomposition of the third layer, and the corresponding wavelet coefficients are shown in Figure 4a. The variables in the high frequency part were found to decrease when the decomposition layer number increases. Therefore, it contained less extracted information and less available information, leading to the decline in discrimination ratio. From the viewpoint of low frequency components, which were handled the same way as the high frequency parts for each decomposition layer, it is shown in Figure 3b that the PLS-DA model showed unsatisfactorily low frequency discrimination performance. The RF and LS-SVM models show low frequency discrimination performance that was slightly inferior to the high frequency discrimination, and the best low frequency discrimination accuracy was still lower than 60%. Meanwhile, discrimination performance largely varied in the different layers, like the high frequency results.

The above analysis shows that an essential role in data compression and information extraction was played by DWT, and DWT can improve the discrimination performance of RF and LS-SVM. However, DWT is merely the time–frequency analysis of spectral signals. It does not extract characteristic information for the identification of the different types of *Tegillarca granosa*. For the variables in the domain of wavelet decomposition, there is irrelevant information that does not contribute to discrimination. Therefore, the approach to eliminate such irrelevant information should be included for better performance. In this study, IGA, a prevalent characteristic data mining method used in classification, was adopted by our group. Based on the previous results, the best discrimination performance was from the data of the third decomposition layer; hence, the high frequency coefficients of the third layer in Figure 4a were further analyzed to extract the characteristic information by the IGA. As shown in Figure 4b, the extracted information was greatly narrowed in comparison with the information of the high frequency of the third layer, and the number of variables was sharply reduced to only 30.

These 30 characteristic variables were set as the input variables of the PLS-DA, LS-SVM, and RF models. For the PLS-DA model, the discrimination result was slightly improved, but the discrimination ratio was still lower than 40%. Compared with the discrimination model based on the variables of the full spectra and the high frequency coefficients of the third layer, the discrimination model built based on the variables extracted with the IGA significantly improved the recognition ratios for the LS-SVM and RF models to 86.7% and 93.3%, respectively. This improvement was primarily attributed to the IGA algorithm’s eliminating the unrelated or useful information for discrimination. With the combination of DWT and IGA methods, the number of variables was obviously reduced from 30,546 in the full spectrum to 30 effective variables, exhibiting a sharp cut rate of 99%, which also saved a considerable portion of computing power for the recognition process.

### 3.4. Analysis of Reconstructed Spectra

**Figure 5:**
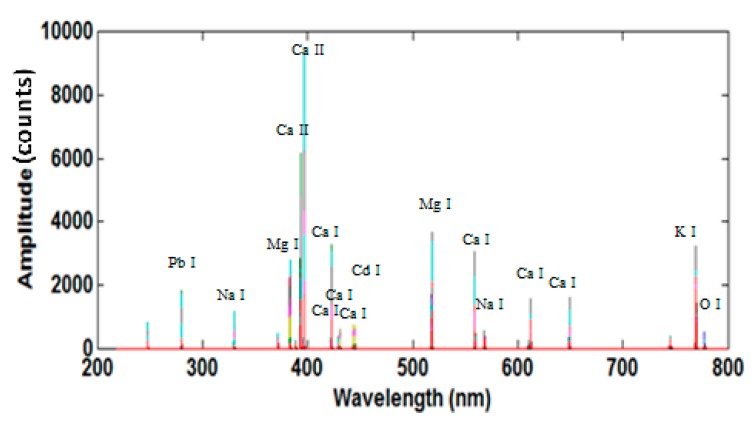
LIBS spectra of Group IV samples after reconstruction by inverse discrete wavelet transform (DWT) using the extracted information.

Since the input variables of the above discrimination models were processed with the DWT algorithm, their coefficients were in the wavelet domain rather than in the original spectra. Therefore, the characteristic variable information could not directly reflect the characteristic element information of the difference between the varied kinds of *Tegillarca granosa* samples. To further analyze the related elemental information, the characteristic information was processed with inverse DWT (IDWT) and reconstructed to the original spectral domain. Figure 5 displays the reconstructed spectra of Group IV samples (i.e., the reconstructed coefficients herein were made by the absolute values). Comparisons between the original and the reconstructed spectra show that the latter contain fewer variables, mainly due to the considerable noise variables and the uninformative variables eliminated by WTA and IGA. In this regard, the corresponding attribution analysis of the spectral peaks for the major characteristic variables was conducted based on the reconstructed spectra. The results are shown in Table 1, where the reconstructed spectra comprise the characteristic peaks of Mg, Ca, P, K, Pb, and Cd.

In the spectra, the spectral lines for Mg and Ca, located near 400 nm, had higher absolute values and made a greater contribution to the discrimination model, while the other Mg and Ca bands contributed less. The characteristic spectral lines of heavy metals, Pb and Cd, contributed even less to the model. Besides, it was also discovered that there were no characteristic spectral lines for Zn found in the reconstructed spectra, probably because the Zn concentration, as one of the essential elements in the body, varied slightly in the heavy-metal-contaminated *Tegillarca granosa* samples. In addition, according to the attributes of the identified spectral lines, the process of heavy metal poisoned *Tegillarca granosa* is very complicated and often accompanied by many protein syntheses, lysosomal changes, and immune damages [45,46,47]. The *Tegillarca granosa*’s normal food intake may be affected in heavy-metal-contaminated water, leading to the varied intake of essential trace elements, including Mg, Ca, and K. However, the characteristic information for spectra collected under different metal contamination conditions describes not only the elements that are concentrated within the body (e.g., Cd and Pb), but also other biologically imperative elements, including Mg, Ca, and K. Therefore, essential trace elements including Mg, Ca, and K within body are also affected by different heavy metals, which provide the characteristic information for rapidly identifying heavy-metal-contaminated *Tegillarca granosa*.

## 4. Conclusions

In summary, a rapid discrimination method of toxic heavy-metal-contaminated *Tegillarca granosa* was investigated in this study by combining LIBS and pattern recognition. LIBS technology was applied to acquire spectral signals, which could be analyzed to determine their contamination categories. A novel combination of the DWT-IGA method was employed to extract the high frequency information and the characteristic information as the input of the discrimination analysis. The performance of discriminating the healthy samples from the toxic samples with three different classifiers was discussed. Results show that the RF model demonstrates the highest elemental discrimination ratio of 93.3% for different types of *Tegillarca granosa*. Our work suggests that LIBS technology combined with pattern recognition analysis can conveniently detect toxic heavy-metal-contaminated shellfish samples, which may induce more promising eco-toxicological testing applications. Moreover, rapid in-field identification of healthy shellfish seafood can also be expected by integrating portable LIBS equipment and machine learning methods, for real-time monitoring and protection of the coastal marine environment.

## Figures and Tables

**Table 1:** Analysis of characteristic spectral band attributes.

Spectral Emission Lines (nm)	Elements
280.2	Pb I
330.1	Na I
383.5	Mg I
393.4	Ca II
396.8	Ca II
428.7	Ca I
430.8	Ca I
443.4	Ca I
445.3	Ca I
467.8	Cd I
518.3	Mg I
558.9	Ca I
568.4	Na I
612.1	Ca I
649.4	Ca I
769.5	K I
777.5	O I

Note: Band attribution according to National Institute of Standards and Technology (http://www.nist.gov/pml/data/handbook/index.cfm). I: atomic spectral lines, II: ionic spectral lines.
